# Integrated transcriptomic and metabolomic analyses reveal distinct energy metabolic signatures and functional properties of RPE cells under two culture conditions

**DOI:** 10.1038/s41598-026-39689-9

**Published:** 2026-04-10

**Authors:** Fan Zhang, Chenlu Wang, Qinxue Tang, Tangyan Ao, Juan Li, Yong Liu

**Affiliations:** 1 Jinfeng Laboratory, Chongqing, 400039 China; 2 Department of Ophthalmology, The First Affiliated Hospital of Army Medical University (Southwest Hospital), Chongqing, China; 3 Chengdu, 610041 China; 4 Building 4, Room 211, 313 Jin Yue Road, Gao Xin District, Chongqing, 400039 China; 5 Building 4, Room 222, 313 Jin Yue Road, Gao Xin District, Chongqing, 400039 China

**Keywords:** Retinal pigment epithelium, Fatty acid oxidation, Glycolysis, Cell biology, Molecular biology, Stem cells

## Abstract

Retinal pigment epithelium (RPE) degeneration is a major cause of vision loss in multiple retinal diseases, including age-related macular degeneration, Bietti crystalline dystrophy and Stargardt disease. Induced pluripotent stem cell (iPSC)-derived RPE cells hold promise for regenerative therapies. B27- and KSR-based media are among the most commonly used for RPE culture; however, how these culture conditions shape RPE cell identity and features remain incompletely understood. Here, we performed comprehensive transcriptomic and metabolomic profiling of iPSC-derived RPE cells cultured in B27 or KSR media to systematically compare their gene expression and metabolic features. B27- and KSR-cultured RPE cells exhibited distinct morphologies and barrier properties. Integrated multi-omics analyses revealed that KSR-cultured RPE cells displayed a relative bias toward fatty acid oxidation and oxidative phosphorylation, whereas B27-cultured RPE cells showed a relative bias toward glycolytic metabolism. The glycolytic tendency observed in B27-RPE cells was accompanied by increased expression of extracellular matrix–related genes and higher transepithelial resistance. In contrast, KSR-RPE cells exhibited comparable tricarboxylic acid cycle activity but higher expression of oxidative phosphorylation–related genes compared with B27-RPE cells. Together, these results demonstrate that RPE cells cultured under different conditions adopt distinct but partially overlapping metabolic and transcriptional states, which are associated with differences in RPE-related features and barrier properties. Our findings highlight the importance of metabolic balance between glycolysis and fatty acid oxidation in shaping in vitro RPE phenotypes.

**Supplementary Information:**

The online version contains supplementary material available at 10.1038/s41598-026-39689-9.

## Introduction

The retinal pigment epithelium (RPE) is a monolayer of pigmented, polarized cells located between the neural retina and the choroid. On the apical side, RPE cells interdigitate with photoreceptor outer segments and play an essential role in maintaining photoreceptor homeostasis and function^1^. For example, the RPE enzymatically isomerizes all-trans-retinol to 11-cis retinal for the visual cycle, phagocytizes photoreceptor outer segments and provides metabolic support for photoreceptors^1,2^. On the basal side, the RPE interfaces the choroid, a vascular structure that supports both the RPE and the outer retina. The choroid supplies oxygen and biomolecular nutrients such as glucose and fatty acids through the vasculature system and is transported by the RPE to the retina^3^. Thus, RPE dysfunction is connected to many ocular disorders and severe vision loss, ranging from inherited retinal disease (IRD) to age-related macular degeneration (AMD)^4^. Although the RPE can be imaged in vivo, human RPE tissue is difficult to obtain for experimental and mechanistic studies. Consequently, it is challenging to fully capture the molecular features underlying RPE-related retinal diseases and to develop effective therapeutic strategies. In this context, human induced pluripotent stem cell (hiPSC)-derived RPE cells provide a valuable in vitro model system and a potential cell source for regenerative therapies.

In vitro cultivation of RPE cells has been developed over several decades, resulting in a wide range of culture protocols that generate cells with varying degrees of similarity to the natural RPE^5^. Primary human and animal RPE cells derived from tissue or cell lines are commonly cultured in DMEM supplemented with fetal bovine serum (FBS) or serum-free medium supplemented with B27^5^. Human ESCs and iPSCs can also be differentiated into RPE cells in knockout medium (KOM) with KO serum replacement (KSR)^6^, which has been applied in clinical trials for RPE transplantation^7^ and RPE-related disease models^8^. For culturing human ESCs and iPSC-derived RPE cells, KSR and B27 are the most widely used additive factors^9–11^. Notably, some RPE features, such as tight junction proteins and transepithelial electrical resistance (TER), differ between hESC-RPE cells cultured in KSR medium and those cultured in B27-based serum-free medium^9^. In addition, a systematic characterization of the molecular and metabolic differences between iPSC-derived RPE cells maintained under these distinct culture conditions remains lacking.

Here, we systematically compared the features of iPSC-derived RPE cells maintained in B27 and KSR media on the basis of comprehensive transcriptomic and metabolomic profiles. Our results show that RPE cells maintained under the two conditions share broadly similar global transcriptomic profiles and express canonical RPE markers, albeit at different levels that are associated with distinct cellular features. Integrated omics analyses revealed that KSR-RPE cells presented relatively higher fatty acid oxidation (FAO) related gene expression and metabolic flux, whereas B27-RPE cells exhibit a relative bias toward glycolytic metabolism. The glycolytic tendency observed in B27-RPE cells is closely accompanied by increased expression of extracellular matrix (ECM) related genes and higher TER. In contrast, KSR- and B27-RPE cells show comparable activity of the tricarboxylic acid cycle, while KSR-RPE cells exhibit higher levels of oxidative phosphorylation. These findings provide a comprehensive analysis of the molecular and metabolic characteristics of in vitro RPE cells, offering valuable insights for their application in disease modeling and cell transplantation. Moreover, they also revealed a close link between RPE metabolism, gene expression, and RPE features such as barrier function.

## Results

### iPSC-derived RPE cells maintained under distinct culture conditions exhibit morphological and barrier functional differences

**Fig. 1:**
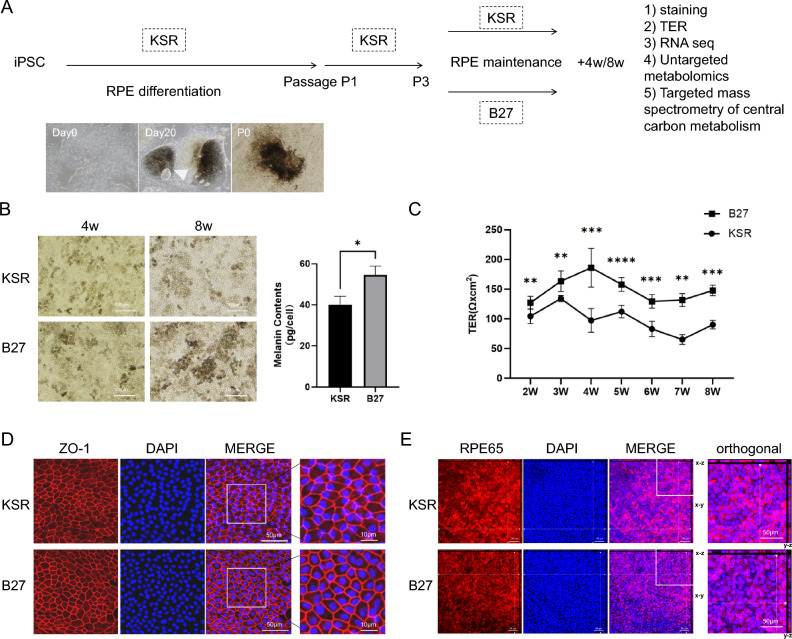
iPSC-derived RPE cells maintained under distinct culture conditions exhibit morphological and barrier functional differences. (**A**) Scheme of differentiation protocol from iPSCs to RPE cells and study design, pigmented foci is indicated with white triangle; (**B**) Morphology of KSR- and B27-RPE cells under white light(left), melanin contents of KSR- and B27-RPE cells at 8 weeks; *P < 0.05, using unpaired *t*-tests; N = 3 per group. Values are expressed as means ± SEM; (**C**) Measurement of transepithelial electrical resistance (TER) of KSR- and B27-RPE cells at different time points; *P < 0.05, **P < 0.01, ***P < 0.001 and ****P < 0.0001, using unpaired *t*-tests; N = 6 per group. Values are expressed as means ± SEM; (**D**) Tight junction marker ZO-1 immunostaining of 4-week cultured KSR- and B27-RPE cells; (**E**) Visual cycle marker RPE65 immunostaining of 4-week cultured KSR- and B27-RPE cells.

A commercially available, healthy donor–derived iPSC line were induced into RPE cells using the nicotinamide method with KSR based differentiation medium^6^. To investigate how culture conditions shape RPE cell identity, we maintained RPE cells under two commonly employed culture conditions: B27-supplemented serum-free medium (referred to as B27 medium) and KSR-based medium (KSR medium), as demonstrated in (Fig. 1A). Compared with KSR-RPE cells, B27-RPE cells showed increased pigment deposition (Fig. 1B) and higher transepithelial electrical resistance (TER) (Fig. 1C). Similar trends of deeper pigment deposition and elevated TER were also observed in RPE cells derived from additional donors cultured in B27 medium (Fig. S1A–C). Consistent with these observations, B27-RPE cells displayed a more regular ZO-1 staining pattern, indicative of well-defined cell boundaries (Fig. 1D). In contrast, the staining of RPE65 was more intense and more accurately localized in the cytoplasm in KSR-RPE cells (Fig. 1E). Both RPE populations retained the ability to phagocytose photoreceptor outer segments (POS) (Fig. S1D). Collectively, these results indicate that iPSC-derived RPE cells cultured in B27 or KSR media exhibit distinct morphological and barrier functional features.

### Transcriptomic profiling reveals divergent gene expression patterns associated with metabolic regulation in RPE cells cultured in B27 and KSR media

**Fig. 2:**
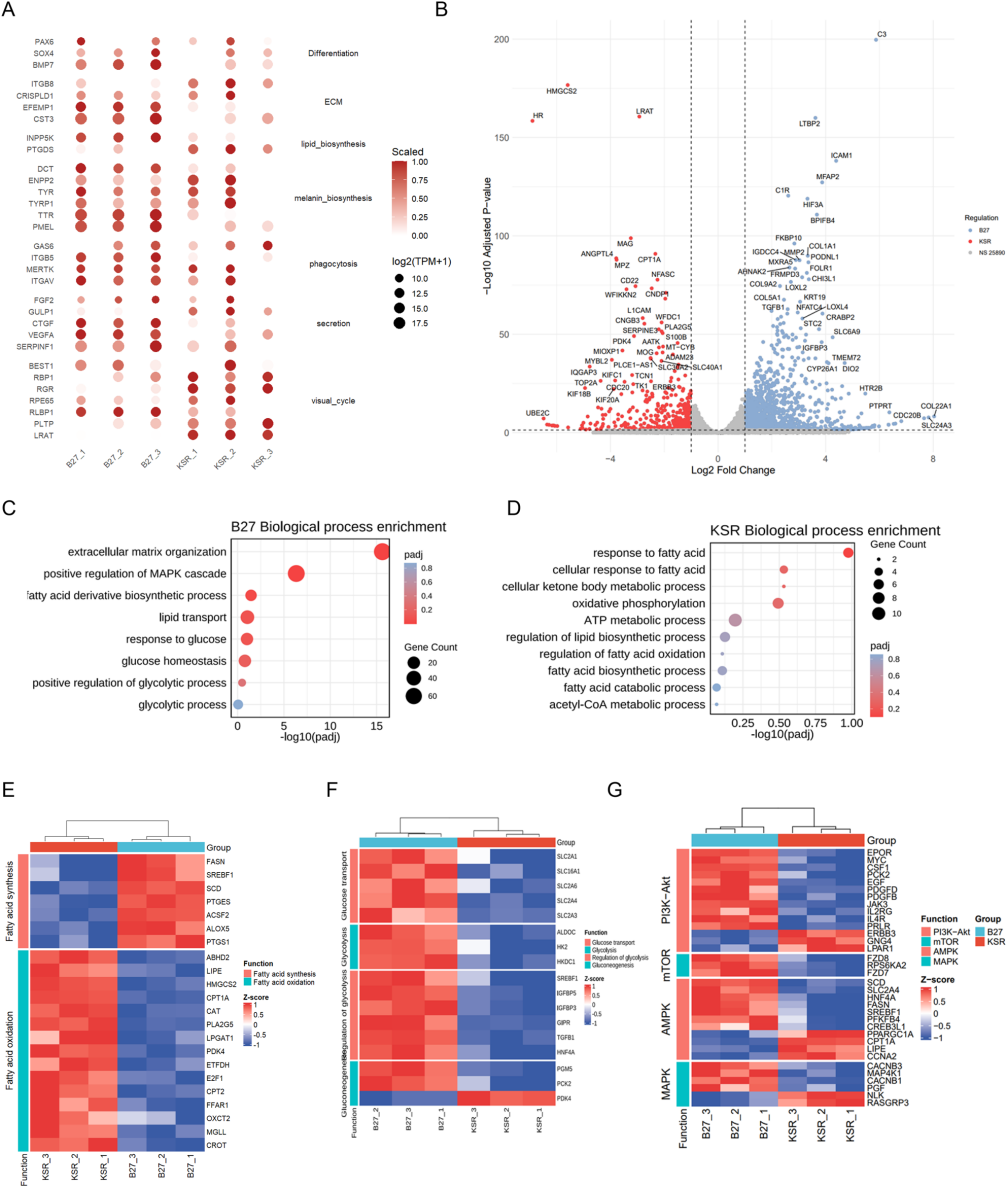
Transcriptomic profiling reveals divergent gene expression patterns associated with metabolic regulation in RPE cells cultured in B27 and KSR media. (**A**) Canonical RPE marker expression in 4-week cultured KSR- and B27-RPE cells; (**B**) Volcano plot of differentially expressed genes between the two groups; (**C**,**D**) GO enrichment analysis of upregulated genes in B27-RPE cells (**C**) and KSR-RPE cells (**D**); E: Heatmap of differentially expressed genes related to “fatty acid metabolism”(**E**), “glucose metabolism” (**F**) and metabolic regulatory pathways (**G**) of KSR- and B27-RPE cells.

To investigate the molecular differences between the two RPE cell populations, we performed transcriptomic analyses of RPE cells cultured in the two media for 4 weeks. Principal component analysis (PCA) revealed consistent clustering of three biological replicates within each group, with B27- and KSR-cultured RPE cells forming two clearly separated clusters (Fig. S2A). Spearman correlation analysis based on global gene expression profiles indicated a high overall similarity between the two groups (Fig. S2B). Both RPE populations robustly expressed canonical RPE markers^12^, including those associated with RPE differentiation (PAX6, SOX4, BMP7), extracellular structure organization (ECM) (CST3, EFEMP1, CRISPLD1, and ITGB8), lipid biosynthesis (PTGDS), melanin biosynthesis (PMEL, TTR, TYR, TYRP1, and DCT), phagocytosis (ITGAV, MERTK, ITGB5 and GAS6), secretion (SERPINF1, VEGFA, CTGF, GULP1 and FGF2) and the visual cycle (RPE65, BEST1, RBP1, RLBP1, RGR, and LRAT) (Fig. 2A). The B27 RPE exhibited relatively higher expression of genes related to ECM and melanin biosynthesis, which is consistent with deeper pigment deposition and increased TER (Fig. 1B and C), and the KSR RPE showed higher expression of visual cycle genes, in agreement with stronger RPE65 immunostaining (Fig. 1E). Collectively, these results indicate that although iPSC-derived RPE cells cultured in B27 or KSR media display highly similar global transcriptomic profiles and canonical RPE identity, they exhibit distinct expression patterns in functional gene modules relevant to RPE features.

Given the overall similarity in global transcriptomic profiles, we next sought to identify specific functional programs that distinguish RPE cells cultured in B27 or KSR media. Differential expression analysis revealed that the majority of genes (25,890) exhibited comparable expression levels between the two groups, whereas 1278 genes were upregulated and 406 genes were downregulated in B27-RPE cells compared with KSR-RPE cells (Fig. 2B and S2C). Gene Ontology (GO) analysis revealed that the upregulated genes of B27-RPE cells were enriched in pathways related to “extracellular matrix organization”, “fatty acid biosynthetic process”, “response to glucose” and “glycolytic process” (Fig. 2C), and the upregulated genes in KSR group were related to “response to fatty acid”, “oxidative phosphorylation” and “fatty acid oxidation” (Fig. 2C). KEGG analysis^13–15^ of the all differentially expressed genes (DEGs) also revealed enriched pathways involved in metabolic regulation, such as the PI3K-Akt, MAPK, AMPK and mTOR signaling pathways (Fig. S2D), which are crucial for regulating key energy metabolism processes such as glycolysis and fatty acid oxidation.

To further characterize differences in metabolic gene expression between the two groups, we examined genes involved in core metabolic pathways related to fatty acid and glucose metabolism based on GO and KEGG annotations. Heatmap analysis (Fig. 2E) revealed that genes related to fatty acid synthesis and storage were relatively upregulated in B27-RPE cells (FASN, SCD, SREBF1, ACSF2). In contrast, KSR-RPE cells exhibited relatively higher expression of genes involved in lipid mobilization and fatty acid oxidation (Fig. 2E), including lipolysis-associated genes (LIPE, MGLL, ABHD2, and PLA2G5), FAO-related genes (CPT1A, CPT2, CROT, ETFDH, PDK4, and CAT), ketone body metabolism–related genes (HMGCS2 and OXCT2), as well as genes implicated in fatty acid transport, sensing, or regulation (FFAR1, LPGAT1, and E2F1). In addition, genes related to glucose metabolism, including glucose transporter genes^16^ (SLC2A1, SLC16A1, SLC2A6, SLC2A4, and SLC2A3), glycolysis-related genes (ALDOC, HK2, HKDC1)^17^, and regulatory genes involved in glucose metabolism (SREBF1, IGFBP5, and IGFBP3), were significantly upregulated in B27-RPE cells (Fig. 2F). Moreover, the key factors of the PI3K-Akt, MAPK, AMPK and mTOR signaling pathways were upregulated in B27 cells (Fig. 2G) and participate in fatty acid synthesis^18^ (SREBF1, HNF4A, CREB3L1) and glucose metabolism^17^ (SLC2A4, PFKFB4, PCK2). In contrast, the genes (CPT1A, LIPE, CCNA2) upregulated in KSR cells (Fig. 2G) are involved in enhancing fatty acid utilization and oxidation^17,19^. Collectively, these transcriptomic data indicate that RPE cells cultured in B27 or KSR media exhibit distinct metabolic gene expression profiles, with B27-RPE cells showing a relative bias toward glycolytic and lipogenic pathways, whereas KSR-RPE cells display a relative enrichment of genes associated with fatty acid oxidation.

### Integrated transcriptomic and metabolomic analyses reveal metabolic features consistent with enhanced fatty acid oxidation in KSR-RPE cells

RPE cells play essential metabolic roles in the retina, including the phagocytosis of photoreceptor outer segments and the provision of metabolic support to adjacent photoreceptors. Consistent with these functions, RPE cells exhibit highly active energy metabolism. Given that transcriptomic analyses revealed extensive differential expression of metabolism-related genes between B27- and KSR-cultured RPE cells, we next performed untargeted metabolomic profiling to further characterize metabolic differences under the two culture conditions. Principal component analysis revealed consistent clustering of six biological replicates within each group (Fig. S3A). The overall distribution of detected metabolites was broadly similar between B27- and KSR-RPE cells (Fig. S3B), indicating a largely shared metabolic landscape. Nevertheless, 807 metabolites were differentially abundant between the two groups, with 498 enriched in the B27 group and 309 enriched in the KSR group (Fig. S3C). Classification of these differential metabolites according to Class III metabolite categories revealed that the most represented classes were “Amino acids, peptides, and analogs”, “Carbohydrates and carbohydrate conjugates”, and “Fatty acids and conjugates” (Fig. S3D). Based on the transcriptomic enrichment of lipid metabolism–related pathways, we further focused our analysis on fatty acid–associated metabolites, including “fatty acids and conjugates” and “fatty acid esters”.

**Fig. 3:**
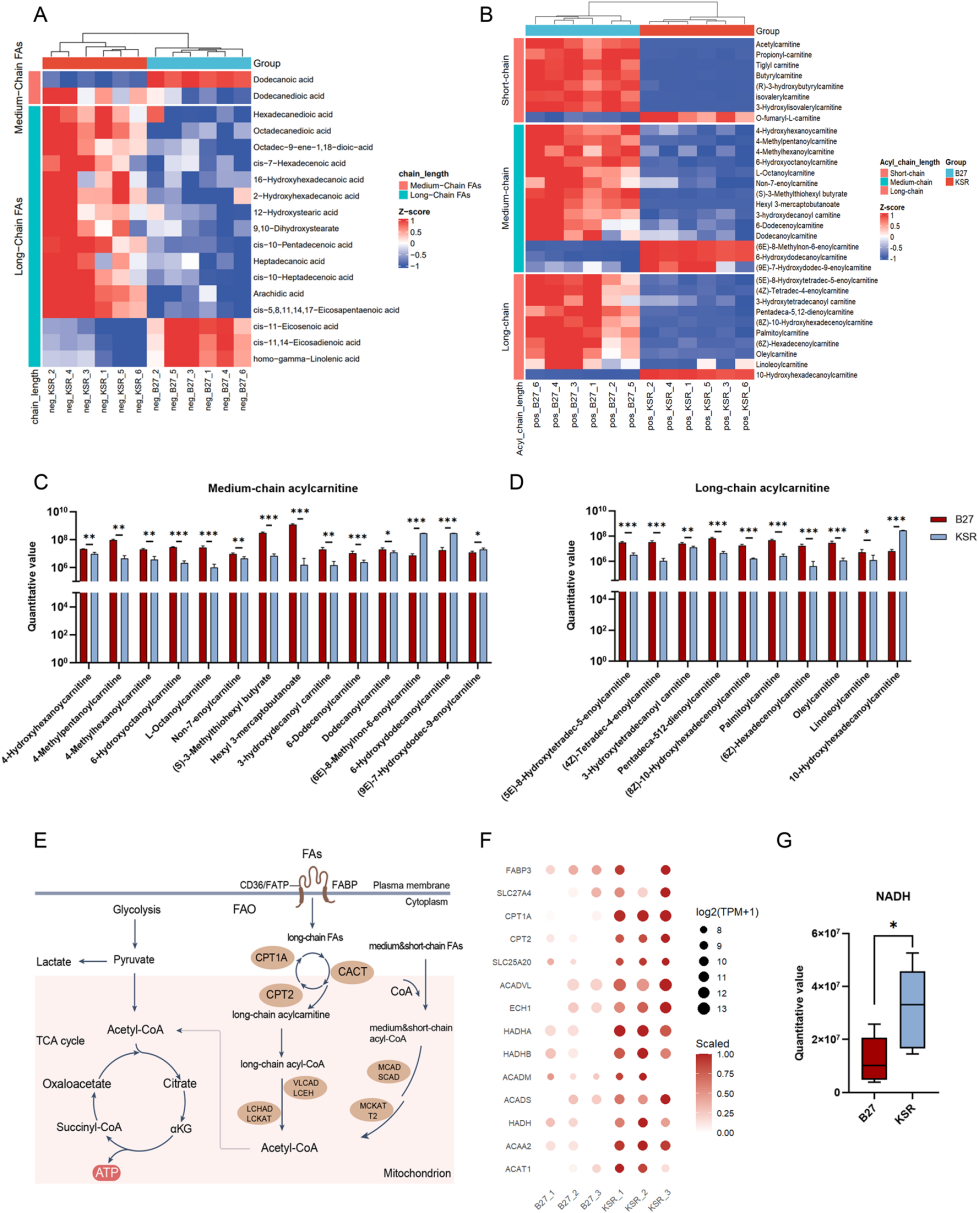
Integrated transcriptomic and metabolomic analyses reveal metabolic features consistent with enhanced fatty acid oxidation in KSR-RPE cells. (**A**) Heatmap of differential abundant long-chain and medium-chain fatty acids involved in mitochondrial β-oxidation of KSR- and B27-RPE cells; (**B**) Heatmap of differential abundant long-chain, medium-chain and short chain acylcarnitine of the two groups; (**C**,**D**) Relative quantification of medium-chain (**C**) and long-chain (**D**) acylcarnitine by untargeted metabolomic mass spectrum; (**E**) Scheme of mitochondrial β-oxidation, involved metabolic enzymes and metabolites; (**F**) Expression of fatty acid mitochondrial β-oxidation metabolic enzymes of the two groups; (**G**) Relative quantification of NADH by untargeted metabolomic mass spectrum; *P < 0.05, using unpaired *t*-tests; N = 6 per group. Values are expressed as means ± SEM.

Medium- and long-chain fatty acids serve as primary substrates for mitochondrial β-oxidation. Within the category of “fatty acids and conjugates”, 18 medium- and long-chain fatty acids were differentially abundant between the two groups, of which 14 were present at higher levels in the KSR-RPE cells (Fig. 3A). In the category of “Fatty acid esters”, a total of 30 differential acylcarnitine metabolites were identified and classified on the basis of the number of carbon atoms in their acyl chains into short-chain (C2–C5), medium-chain (C6–C12), and long-chain (C13–C20) acylcarnitines^19^. Among them, there were 9 short-chain, 11 medium-chain, and 10 long-chain acylcarnitines (Fig. 3B). Among these metabolites, 25 acylcarnitines were relatively increased in the B27 group, whereas only 5 were higher in the KSR group (Fig. 3B). The levels of most medium- and long-chain acylcarnitines in the KSR group were significantly lower than those in the B27 group (Fig. 3C,D). Acylcarnitines are essential intermediates in the process of fatty acid oxidation, facilitating the transport of fatty acids from the cytosol into the mitochondria for β-oxidation^19,20^ (Fig. 3E). Since long-chain fatty acids cannot directly cross the inner mitochondrial membrane, they are first activated in the cytosol to form acyl-CoA^21^, which is then converted into acylcarnitines by carnitine palmitoyltransferase 1 (CPT1). Once transported into the mitochondrial matrix, acylcarnitines are reconverted into acyl-CoA by CPT2 to enter the β-oxidation pathway^21^ (Fig. 3E). Consistent with the elevated levels of free fatty acids observed in the KSR group, KSR-RPE cells exhibited higher expression of fatty acid transport–related genes, including FABP3 and FATP4 (SLC27A4) (Fig. 3F). In addition, key enzymes involved in mitochondrialβ-oxidation, such as CPT1A, CPT2, ACADVL and ACADM^17,21^, also presented higher expression in the KSR group (Fig. 3F). The reduced accumulation of acylcarnitines in KSR-RPE cells (Fig. 3B) is consistent with more efficient mitochondrial utilization of fatty acids. Furthermore, levels of NADH, a major reducing equivalent generated during fatty acid oxidation^21^, were elevated in KSR-RPE cells (Fig. 3G). Collectively, the integrated transcriptomic and metabolomic data indicate that RPE cells cultured in KSR medium exhibit metabolic features consistent with enhanced fatty acid uptake and mitochondrial fatty acid oxidation, whereas B27-RPE cells show relative accumulation of acylcarnitine intermediates, suggesting differences in lipid utilization between the two culture conditions.

### Increased glycolysis is associated with increased ECM gene expression and epithelial barrier characteristics in cultured B27 RPE cells

**Fig. 4:**
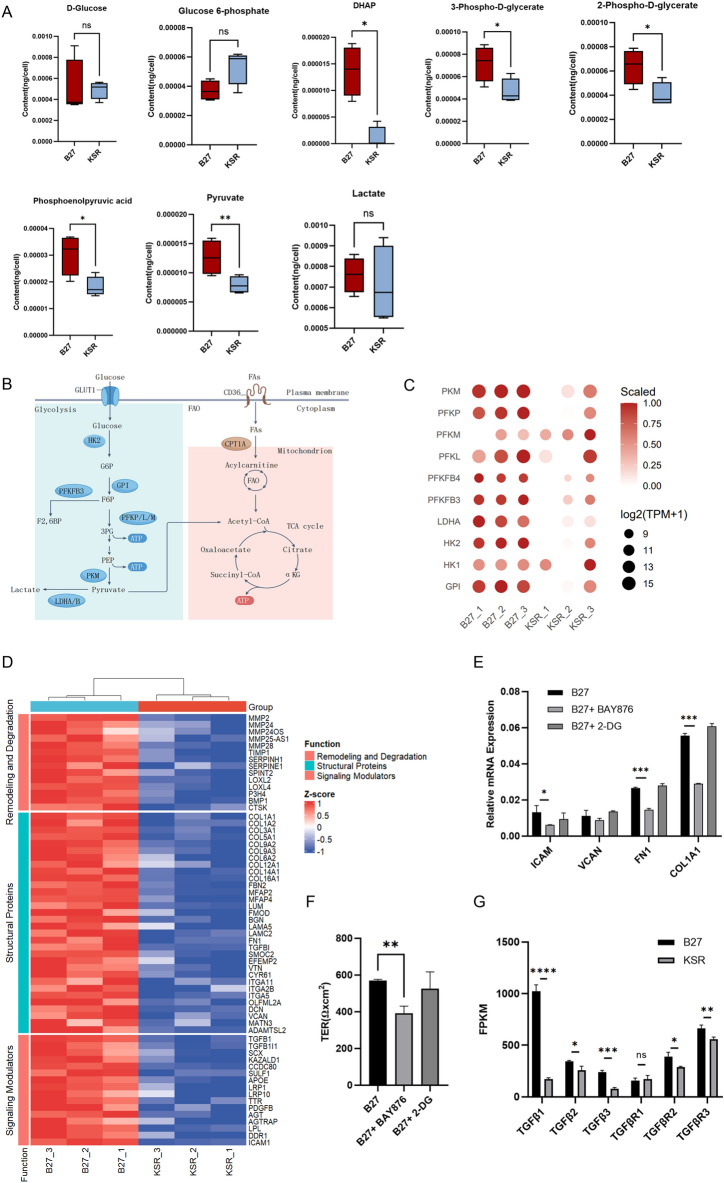
Increased glycolysis is associated with increased ECM gene expression and epithelial barrier characteristics in cultured B27 RPE cells. (**A**) Absolute quantification of glycolysis related metabolites in KSR- and B27-RPE cells by targeted metabolomic mass spectrum; (**B**) Scheme of glycolysis, involved metabolic enzymes and metabolites; (**C**) Expression of glycolysis metabolic enzymes of the two groups; (**D**) Heatmap of differentially expressed genes related to extracellular matrix of KSR- and B27-RPE cells; (**E**) Quantitative PCR of extracellular matrix genes ICAM, VCAN, FN1, and COL1A1 of control (DMSO treated), BAY876 (5uM) inhibited and 2-DG (10 mM) inhibited B27-RPE cells for 2 weeks; (**F**) Measurement of transepithelial electrical resistance of control, BAY876 inhibited and 2-DG inhibited B27-RPE cells for 2 weeks; (**G**) Histograms representing TGFβ1, TGFβ2, TGFβ3, TGFβR1, TGFβR2, and TGFβ3 gene FPKM in KSR- and B27-RPE cells; *P < 0.05, **P < 0.01, ***P < 0.001 and ****P < 0.0001, using unpaired *t*-tests; N = 3 per group. Values are expressed as means ± SEM.

Transcriptomic analysis revealed elevated expression of glycolysis-related genes in the B27 RPE; however, owing to the limitations of the non-targeted metabolomics approach, key glycolytic intermediates such as lactate and pyruvate were not detected. Therefore, we performed targeted metabolomics of central carbon metabolites to measure glycolytic products. Quantitative results showed that glucose levels were comparable between B27 and KSR RPE, and glucose-6-phosphate levels were slightly higher in the KSR group without significant (Fig. 4A,B). The levels of glycolytic intermediates^17^, including dihydroxyacetone phosphate (DHAP), 3-phospho-D-glycerate (3PG), 2-phospho-D-glycerate (2PG), phosphoenolpyruvic acid (PEP), and pyruvate in B27 RPE cells, were significantly higher than those in KSR cells although lactate levels were not significantly different between two groups (Fig. 4A,B). Lactate abundance represents a steady-state balance between production, utilization, and transport and does not necessarily reflect glycolytic pathway activity. In B27-RPE cells, alanine-associated metabolites were increased (Fig. S4A), indicating that a fraction of pyruvate may be directed toward alanine-related metabolic pathways rather than conversion to lactate. Consistently, lactate levels remained unchanged despite the accumulation of multiple upstream glycolytic intermediates in B27-RPE cells. Consistent with these metabolic differences, the expression of key glycolytic enzymes^22,23^, such as HK2, PFKFB3, PKM and LDHA, was markedly higher in the B27 group (Fig. 4C). In addition, transcriptomic analysis revealed differential expression of genes involved in upstream signaling pathways regulating glucose metabolism. Components of the PI3K–AKT pathway^24^, including MYC, PCK2, PDGFB, EGF, and IL4R, were relatively upregulated in B27-RPE cells (Fig. 2G), which is consistent with increased expression of glucose transporters such as SLC2A1 (GLUT1) and glycolytic enzymes such as HK2. Together, these transcriptional and metabolomic data indicate that B27- and KSR-RPE cells adopt distinct metabolic states, with B27-RPE cells exhibiting a relative bias toward glycolysis and lipid biosynthesis, and KSR-RPE cells showing transcriptional features consistent with enhanced fatty acid oxidation.

RPE cells cultured under these distinct metabolic states also displayed differences in cellular and epithelial characteristics. B27-RPE cells exhibited a more regular morphology with clearer cell boundaries (Fig. 1D), higher expression of ECM proteins, and higher TER (Fig. 1C and Fig. 2A). Consistently, transcriptomic profiling revealed elevated expression of ECM-related genes in B27-RPE cells (Fig. 4D), suggesting a potential association between cellular metabolic state and ECM remodeling. Previous studies have shown that increased glycolysis and reduced fatty acid oxidation are associated with enhanced ECM production in fibrotic tissues^25^. To explore whether glycolytic activity contributes to ECM gene expression in B27-RPE cells, we pharmacologically inhibited glycolysis using 2-deoxy-D-glucose (2-DG) and BAY-876 (Fig. S4B). Inhibition of glycolysis resulted in reduced expression of ECM-related genes, including FN1, ICAM1, COL1A1, and VCAN (Fig. 4E), accompanied by a decrease in TER (Fig. 4F and S4C). In addition, transforming growth factor β1 (TGF-β1), a key regulator of ECM production acting in part through HIF-1α and SMAD signaling^25^, was expressed at higher levels in B27-RPE cells (Fig. 4G). Collectively, these results indicate that RPE cells cultured in B27 medium display a glycolysis-biased metabolic profile that is closely associated with enhanced ECM gene expression and epithelial barrier properties, suggesting a link between metabolic state and RPE functional characteristics.

### KSR-RPE cells exhibit comparable TCA cycle profiles and enhanced oxidative phosphorylation–associated features relative to B27-RPE cells

**Fig. 5:**
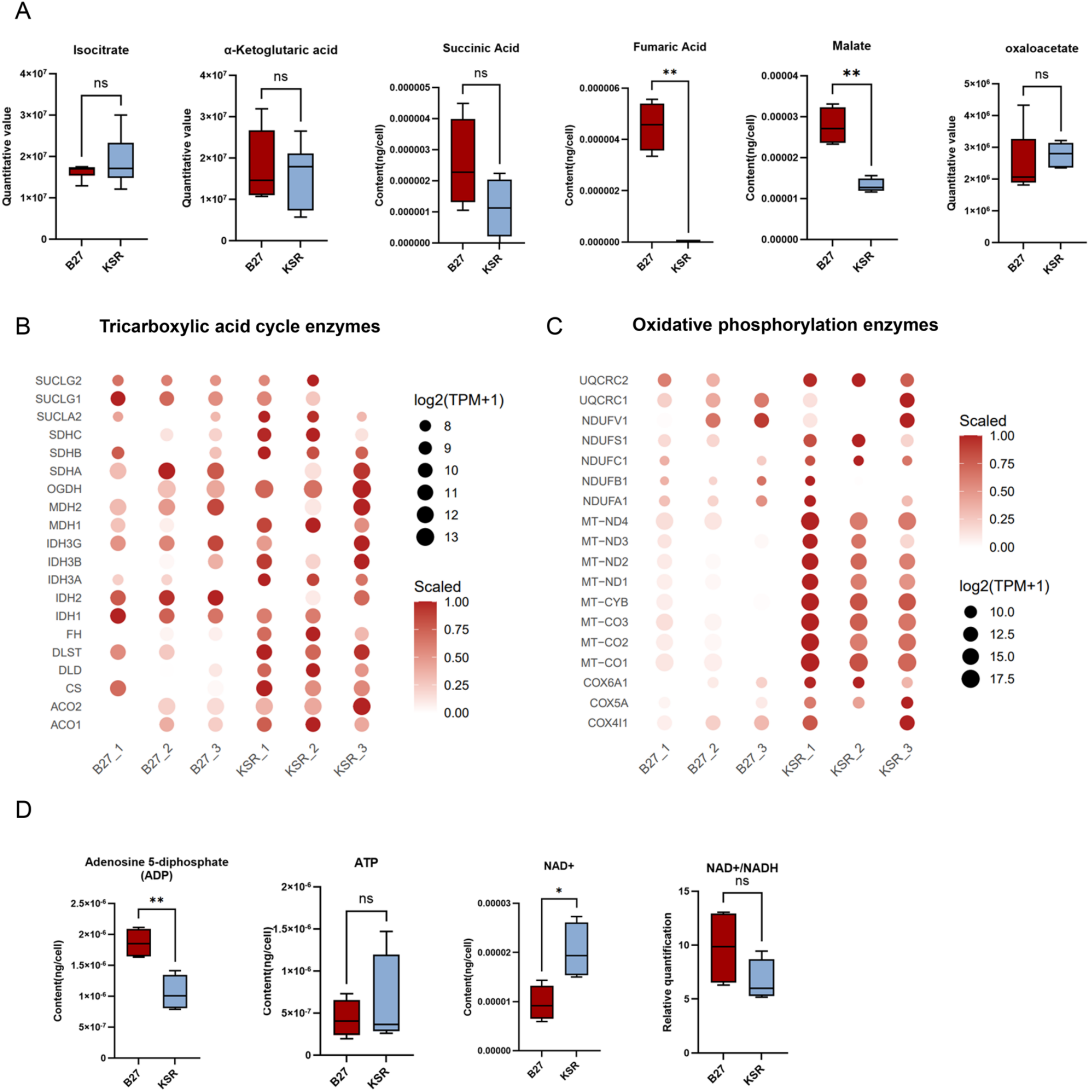
KSR-RPE cells exhibit comparable TCA cycle profiles and enhanced oxidative phosphorylation associated features relative to B27-RPE cells. (**A**) Absolute quantification of TCA related metabolites in KSR- and B27-RPE cells by targeted metabolomic mass spectrum; (**B**,**C**) Expression of TCA cycle (**B**) and oxidative phosphorylation (**C**) metabolic enzymes of the two groups; (**D**) Absolute quantification of ADP, ATP and NAD + by targeted metabolomic mass spectrum, and the ratio of NAD + and NADH in KSR- and B27-RPE cells; *P < 0.05, **P < 0.01, using unpaired t-tests; N = 4 per group. Values are expressed as means ± SEM.

Glycolysis and fatty acid oxidation are two major pathways that generate acetyl-CoA, which enters the tricarboxylic acid (TCA) cycle. The TCA cycle produces reducing equivalents NADH and FADH₂, which fuel oxidative phosphorylation to generate ATP^26,27^. Targeted metabolomics of central carbon metabolism revealed multiple key TCA cycle metabolites in both B27- and KSR-RPE cells. There was no significant difference in isocitrate levels between the B27 and KSR groups, although α-ketoglutarate (α-KG) and succinic acid were slightly elevated in the B27 group, but the difference was not significant (Fig. 5A). Fumaric acid and malate levels were greater in the B27 group than in the other groups, and oxaloacetate levels remained comparable between the two groups (Fig. 5A). Transcriptomic analysis revealed that the expression of key enzymes involved in the TCA cycle was largely similar between the two groups (Fig. 5B). Together, the comparable steady-state levels of most TCA cycle intermediates and the similar expression of TCA-related enzymes suggest that the overall TCA cycle profile is broadly comparable between the two groups, although certain intermediates may accumulate to a greater extent in B27-RPE cells.

Notably, genes encoding oxidative phosphorylation enzymes^27^ were significantly upregulated in the KSR group compared with the B27 group (Fig. 5C). Consistent with this transcriptional pattern, NAD⁺ and NADH levels were both elevated in the KSR group^27^ (Fig. 3G and 5D), while the NAD⁺/NADH ratio was modestly lower than that in the B27 group (Fig. 5D), suggesting increased availability of reducing equivalents. In addition, metabolomic profiling revealed higher ADP levels in B27-RPE cells, whereas ATP levels were slightly higher in KSR-RPE cells, although the difference was not statistically significant (Fig. 5D), consistent with relatively efficient energy conversion under KSR culture conditions. Collectively, these data suggest that KSR-RPE cells exhibit transcriptional and metabolic features consistent with enhanced oxidative phosphorylation compared with B27-RPE cells. This oxidative profile aligns with the increased fatty acid oxidation observed in KSR-RPE cells, which may contribute to elevated NADH availability and support mitochondrial energy metabolism. Taken together, RPE cells cultured in KSR medium display a relative shift toward an oxidative metabolic state, characterized by enhanced fatty acid utilization and oxidative phosphorylation–associated features.

## Discussion

Multiple factors influence the features of RPE cells, including donor origin, the state of the iPSCs or ESCs, the differentiation method, and the maintenance medium. In this study, we cultured RPE cells derived from a commercial wild type iPSC cell line in KSR and B27 media respectively. Although both conditions supported the expression of canonical RPE markers, distinct cellular features were observed. B27-RPE cells presented higher tight junction protein expression, greater TER and deeper pigment deposition, whereas KSR-RPE cells presented stronger visual cycle protein expression like RPE65. Similar culture-dependent differences in epithelial properties, including tight junction protein expression and TER, have also been reported in hESC-derived RPE cells cultured in KSR- or B27-based media^9^. Culture condition–dependent variability is not unique to RPE cells but has been widely documented in other cell types used for cell therapy. For example, bone marrow–derived mesenchymal stromal cells (BM-MSCs) cultured in different commonly used media display marked differences in clonogenicity, proliferation, differentiation propensity, and immunomodulatory capacity^28^. Likewise, comparative studies of serum-containing and serum-free media for human T-cell expansion have shown that serum-free conditions often support enhanced proliferation and altered immune phenotypes^29^. These observations highlight that culture media composition—including serum replacement and xeno-free components—can substantially shape cellular states and functional properties.

ESC- and iPSC-derived RPE cells are increasingly used both as in vitro disease models and as candidate cell sources for transplantation in retinal disorders. However, when applied as disease models, there is currently no unified standard for RPE culture conditions. As a result, different laboratories often employ distinct media formulations, which may contribute to divergent observations and interpretations of disease-related phenotypes^30–32^. Beyond disease modeling, ESC- and iPSC-derived RPE transplantation represents the most advanced application of retinal cell therapy to date. Multiple clinical trials targeting non-exudative age-related macular degeneration (AMD), Stargardt disease, and retinitis pigmentosa are currently underway^33^. Autologous iPSC-derived RPE transplantation has been shown to be safe and feasible, exemplified by the first successful iPSC-derived RPE sheet transplantation performed in Japan^34^. Although transplanted RPE cells are routinely evaluated for safety and characterized by RPE-specific attributes, the molecular and metabolic features that define optimal transplantable RPE cells have not been uniformly established across studies^7,35,36^. As efforts advance toward HLA-matched or allogeneic RPE transplantation, systematic evaluation and standardization of RPE characteristics relevant to engraftment, survival, and functional performance will be increasingly important. In this context, integrated transcriptomic and metabolomic profiling offers a powerful framework for defining RPE cell states beyond traditional marker-based assessments.

Physiologically, RPE cells participate in a tightly coupled metabolic ecosystem with photoreceptors. Glucose derived from the choroidal circulation is transported across the RPE to photoreceptors, which preferentially metabolize glucose through aerobic glycolysis and release lactate. This lactate is subsequently taken up by RPE cells and utilized for oxidative phosphorylation, thereby suppressing glucose consumption within the RPE^37,38^. Disruption of this metabolic compartmentalization—such as reduced lactate availability following photoreceptor degeneration—has been shown in animal models to induce increased glucose utilization by RPE cells. Owing to the limited availability of human RPE tissue, most insights into RPE metabolic specialization are derived from mouse or Xenopus laevis models, and direct metabolomic characterization of human in vivo RPE remains challenging.

In the present study, RPE cells cultured in KSR or B27 media exhibited distinct but overlapping metabolic states. KSR-RPE cells displayed transcriptional and metabolic features associated with enhanced fatty acid oxidation and oxidative phosphorylation, whereas B27-RPE cells exhibited a relative bias toward glycolysis accompanied by increased extracellular matrix expression and epithelial barrier properties. Importantly, both metabolic programs coexist within RPE cells, and neither culture condition fully recapitulates the in vivo metabolic environment. Instead, these findings suggest that commonly used culture conditions bias RPE cells toward different metabolic and functional states, each capturing specific aspects of native RPE biology. Recognizing and accounting for these metabolic biases will be critical for interpreting in vitro disease models and for optimizing RPE cell preparations for therapeutic applications.

## Materials and methods

### Ethical statement

The human iPSC lines used in this study were purchased from Anhui Shownin Biotechnology, Catalog No. RC01001, China. Informed consent was obtained from the donor according to the provider, and ethical approval was granted by the provider’s institutional review board. Therefore, no additional ethical approval was required for this study.

### iPSC culture

The hiPSC cell line (RC01001) used in this study was derived from healthy human umbilical cord blood cells via a nonintegrating reprogramming method. RC01001 exhibits a clonogenic morphology and gene expression highly similar to that of embryonic stem cells (hESCs), maintains a normal karyotype over the long term, and possesses full pluripotent differentiation potential both in vitro and in vivo. The hiPSC cell line was cultured in Matrigel (Corning)-coated dishes in NcTarget™ hPSC medium (Shownin Bio, RP01020), with the media changed daily and passaged every 3–5 days.

### Differentiation and culture of iPSC-derived RPE cells

hiPSC differentiation into RPE cells was carried out in dishes coated with Matrigel. The sets were replenished with regular media supplemented with NcTarget™ hPSC medium for up to 5 d until the hiPSC colonies reached ~ 90–100% confluence and then transitioned to KSR medium consisting of 78 ml of knockout DMEM (Gibco 10829018, + 4.5 g/L D-Glucose, -L-Glutamine, + Sodium Pyruvate), 200 µl of 2-mercaptoethanol (Invitrogen), 1 ml of NEAA (Gibco), 20 ml of knockout serum replacement (Gibco), 1 ml of GlutaMAX (Invitrogen) and 10 mM nicotinamide per 100 ml. The cells were maintained on a twice-weekly media replenishment regimen with KSR medium. RPE cells appear in culture as distinct pigmented foci that are visible to the naked eye at 3–4 weeks and continue to expand in diameter. At the 6th week, RPE foci were manually isolated via sterile syringe needles, dissociated into small pieces and plated in dishes coated with Matrigel. The cells were maintained on a two-week media replenishment regimen of KSR medium until they formed a confluent, pigmented cell sheet with a cobblestone morphology and were passaged every 3–4 weeks. Before initiating the comparative experiments between the two culture conditions, RPE cells were maintained in KSR medium. All the experiments in the present study were performed with P2-P4 iPRE cultured in permeable polyester membrane inserts (Transwell, Corning, 0.4 μm pore size) coated with Matrigel (Corning). The cells were maintained in KSR medium or B27 medium consisting of 40 ml of DMEM (Gibco C11995500BT, + 4.5 g/L D-Glucose, + L-Glutamine, + 110 mg/L Sodium Pyruvate) and 60 ml DMEM/F-12 (Gibco C11330500BT, + 3.1 g/L D-Glucose, + L-Glutamine, + 15 Mm HEPES) supplemented with B27 (Invitrogen) immediately after passage for the KSR-RPE or B27-RPE.

### Immunofluorescence microscopy

iRPEs on transwells were fixed with 4% paraformaldehyde in 0.1 M phosphate buffer for 30 min, washed with PBS and permeated with 0.2% Triton for 20 min. The iRPEs were blocked with blocking buffer for 1 h and incubated with primary antibodies (ZO-1, Proteintech, 21773-1-AP; RPE 65, Abcam, ERP22579-44) overnight at 4 °C. The samples were subsequently washed with PBST, incubated with secondary antibodies against Alexa 488 (Molecular Probes, CAT# A11029) or Cy3 (Jackson ImmunoResearch, CAT#111-165-144), washed and stained with DAPI. The samples were mounted in fluorescence mounting medium, and the fluorescence localization was visualized with a Zeiss laser scanning confocal microscope.

### Quantification of melanin content

Melanin content in iPSC-derived RPE cells was quantified using a spectrophotometric assay. Briefly, RPE cells cultured under different conditions were washed twice with cold PBS and harvested at the indicated time points. Cell pellets were resuspended in 1 M NaOH containing 10% DMSO and incubated at 80 °C for 1 h to solubilize melanin. After centrifugation to remove insoluble debris, the absorbance of the supernatant was measured at 405 nm using a microplate reader. Synthetic melanin (Sigma-Aldrich) dissolved in the same NaOH/DMSO solution was used to generate a standard curve. Total melanin content was normalized to cell number and expressed as pg melanin per cell.

### Transepithelial electrical resistance (TER)

RPE barrier integrity was assessed by measuring transepithelial electrical resistance. RPE cells were seeded on permeable polyester membrane inserts coated with Matrigel. The TER was measured via an epithelial voltohmmeter (EVOM2), and blank inserts containing only medium were used as controls. Resistance values (Ω) were recorded from both the apical and basal chambers and corrected by subtracting the background resistance of the blank inserts. The final TER values (Ω·cm^2^) were calculated by multiplying the net resistance by the surface area of the insert membrane.

### Photoreceptor outer segment (POS) phagocytosis assay

Photoreceptor outer segments (POS) were isolated from fresh porcine retinas obtained from a local abattoir. Briefly, retinas were dissected under dim red light and POS were purified by sucrose gradient centrifugation. Purified POS were labeled with fluorescein isothiocyanate (FITC) and stored at − 80 °C until use. Prior to the phagocytosis assay, POS were thawed on ice and resuspended in serum-free culture medium. RPE cells were cultured for 4 weeks until formation of a mature polarized monolayer. FITC-labeled POS were added to RPE cells and incubated at 37 °C for 4 h to allow phagocytosis. After incubation, cells were washed extensively with PBS to remove unbound POS. Cells were fixed with 4% paraformaldehyde, nuclei were counterstained with DAPI and ZO-1 immunofluorescence was used to define intercellular junctions. Images were acquired using a confocal microscope. Phagocytic activity was quantified as intracellular FITC fluorescence intensity per cell using ImageJ software.

### RNA isolation and reverse transcription PCR

Total RNA was extracted from the iPRE maintained in KSR or B27 medium for 4 weeks via TRIzol™ Reagent (Invitrogen), reverse transcribed into cDNA with ReverTra Ace qPCR RT Master Mix (TOYOBO, FSQ-301) and amplified with ChamQ Universal SYBR qPCR Master Mix (Vazyme, Q711-02-AA). All the samples were treated with DNase. The relative expression of the target mRNAs was normalized to that of GAPDH. The gene-specific primers used are listed in Supplementary Table 1. Data obtained from 3 samples from each group were used for quantitative analysis with GraphPad Prism 8, and P values were calculated via unpaired two-tailed Student’s t tests.

### RNA-seq analysis

#### Sample collection and preparation

RNA integrity was assessed via the RNA Nano 6000 Assay Kit of the Bioanalyzer 2100 system (Agilent Technologies, CA, USA). Total RNA from iRPE cells cultured for 4 weeks was used as input material for RNA sample preparation. Briefly, mRNA was purified from total RNA via poly-T oligo-attached magnetic beads. Fragmentation was carried out using divalent cations under elevated temperature in First Strand Synthesis Reaction Buffer (5X). First-strand cDNA was synthesized via random hexamer primers and M-MulV reverse transcriptase (RNase H-). Second-strand cDNA synthesis was subsequently performed via DNA polymerase I and RNase H. The remaining overhangs were converted into blunt ends via exonuclease/polymerase activities. After adenylation of the 3’ ends of the DNA fragments, adaptors with hairpin loop structures were ligated to prepare for hybridization. To preferentially select cDNA fragments 370–420 bp in length, the library fragments were purified with the AMPure XP system (Beckman Coulter, Beverly, USA). PCR was performed with Phusion High-Fidelity DNA polymerase, universal PCR primers and Index (X) Primer. Then, the PCR products were purified, and library quality was assessed on an Agilent Bioanalyzer 2100 system. Clustering of the index-coded samples was performed on a cBot Cluster Generation System via the TruSeq PE Cluster Kit v3—cBot—HS (Illumina). After cluster generation, the library preparations were sequenced on an Illumina NovaSeq platform, and 150 bp paired-end reads were generated.

#### ***Data ***analysis

Raw data in fastq format were first subjected to quality control through in-house Perl scripts. Clean high-quality data were then mapped to the reference genome, the index of the reference genome was built via HISAT2 v2.0.5, and paired-end clean reads were aligned to the reference genome via HISAT2 v2.0.5. The quantification of the gene expression level featureCounts v1.5.0—p3 was used to count the number of reads mapped to each gene. The FPKM value of each gene was calculated on the basis of the length of the gene and the number of reads mapped to that gene. Differential expression analysis was performed via the DESeq2 R package (1.20.0). The resulting P values were adjusted via Benjamini and Hochberg’s approach for controlling the false discovery rate. Genes with an adjusted P value < 0.05 according to DESeq2 were considered differentially expressed, and the differentially expressed genes are listed in Supplementary Table 2. Gene Ontology (GO) enrichment analysis of differentially expressed genes was implemented via the clusterProfiler R package, in which gene length bias was corrected. GO terms with corrected P values less than 0.05 were considered significantly enriched with DEGs. The enrichment analysis of DEGs via the Kyoto Encyclopedia of Genes and Genomes (KEGG) database was performed via the clusterProfiler R package.

### Targeted metabolomics of central carbon metabolism

#### Metabolite extraction and the LC‒MS method

For standard solution preparation, a stock solution of individual CCM-related compounds was mixed and prepared in a CCM-related compound-free matrix to obtain a series of calibrators. Certain concentrations of D-glucose-13C6, (s)-malic acid-D3, succinic acid-D4, dAMP-lithium salt-15N5 and D-glucose-6-phosphate disodium salt-13C6 were compounded and mixed as internal standards (ISs). For metabolite extraction, the collected iRPEs were centrifuged at 1000 rpm for 3 min (4 °C) to remove the supernatant, homogenized with 250 μL of methanol (80%), which contained mixed internal standards, and centrifuged at 15,000 rpm for 15 min (4 °C) to remove the protein. The supernatant was added to water by vortexing as the diluted sample. Then, 100 μL of each sample was homogenized with 100 μL of imino-bis (methylphosphonic acid) by vortexing, and the mixture was centrifuged at 15,000 rpm for 15 min. Finally, the supernatant was injected into the LC‒MS/MS system for analysis. An ultrahigh-performance liquid chromatography coupled with tandem mass spectrometry (UHPLC-MS/MS) system (ExionLC™ AD UHPLC-QTRAP 6500 + , AB SCIEX Corp., Boston, MA, USA) was used to quantify CCM-related compounds at Novogene Co., Ltd. (Beijing, China). Separation was performed on a Waters Atlantis Premier BEH Z-HILIC column (2.1 × 100 mm, 1.7 μm), which was maintained at 50 °C. The mobile phase, consisting of 15 mM ammonium acetate with 10 μm imino-bis (methylphosphonic acid) (solvent A) and 15 mM ammonium acetate/acetonitrile (solvent B), was delivered at a flow rate of 0.40 mL/min. The solvent gradient was set as follows: initial 95% B, 5 min; 95–70% B, 8 min; 70–40% B, 16 min; 40% B, 21 min; 40–95% B, 22.1 min; 95% B, 24 min. The mass spectrometer was operated in negative multiple reaction mode (MRM) mode. The parameters used were as follows: ion spray voltage (-4500 V), curtain gas (35 psi), ion source temperature (550 °C), and ion source gases of 1 and 2 (60 psi).

#### Quantification and analysis

LC‒MS was used to detect the concentration series of the standard solutions. The ratio of the concentration of the standard to the internal standard was used as the abscissa, and the ratio of the peak area of the standard to the internal standard was used as the ordinate to investigate the linearity of the standard solution. A correlation coefficient (r) > 0.99 for each metabolite was the necessary condition. The limit of quantification (LOQ) was determined by the signal-to-noise ratio (S/N), which compares the signal measured by the standard solution concentration with that of the blank matrix. Generally, when S/N = 10:1, the corresponding concentration is the LOQ. In terms of quantification, matrix effects, precision, accuracy, and 24-h metabolite stability were thoroughly evaluated and within the acceptable criteria. QC samples were injected at regular intervals throughout each batch. The stability of the method was assessed by calculating the relative standard deviation (RSD) of all the QC samples on the basis of the concentrations of the standard compounds. An RSD ≤ 15% was considered indicative of a stable and reliable method. The quantification results of the central carbon metabolism compounds are listed in Supplementary Table 3. The quantification data obtained from 4 independent samples for each group were used for quantitative analysis with GraphPad Prism8, and p values were calculated via unpaired two-tailed Student’s t tests.

### Untargeted metabolomics

#### Metabolite extraction and the LC‒MS method

The RPE cells were resuspended in prechilled 80% methanol by vortexing. The samples were subsequently melted on ice and allowed to warm for 30 s. After the samples were sonicated for 6 min, they were centrifuged at 5000 rpm and 4 °C for 1 min. The supernatant was freeze-dried, dissolved in 10% methanol and injected into the UHPLC‒MS/MS system for analysis. UHPLC‒MS/MS analyses were performed via a Vanquish UHPLC system (Thermo Fisher) coupled with an Orbitrap Q ExactiveTM HF mass spectrometer at Novogene Co., Ltd. (Beijing, China). The samples were injected onto an ACQUITY UPLC BEH amide column (100 × 2.1 mm, 1.7 μm) via a 12-min linear gradient at a flow rate of 0.2 mL/min. The eluents for the positive and negative polarity modes were eluent A (5 mM ammonium acetate in 90% ACN) and eluent B (5 mM ammonium acetate in 50% ACN). The solvent gradient was set as follows: 2% B, 1.5 min; 2–100% B, 7 min; 100% B, 9 min; 100–2% B, 9.1 min; and 2% B, 12 min. A Q ExactiveTM HF mass spectrometer was operated in positive/negative polarity mode with a spray voltage of 3.5 kV, a capillary temperature of 320 °C, a sheath gas flow rate of 35 arb and an aux gas flow rate of 10 arb, an S-lens RF level of 60, and an aux gas heater temperature of 350 °C.

#### Data analysis

The data files generated by UHPLC‒MS/MS were processed by XCMS to perform peak alignment, peak picking, and quantitation for each metabolite. Then, based on the adduct ions and setting the mass deviation to 10 ppm, a comparison was made between these data and the self-built high-quality secondary spectrum database (NovoMetDB) to obtain results for metabolite identification. After background ions were eliminated from the blank samples, the original quantitative results were normalized via the following formula to obtain the relative peak areas: relative peak areas = raw quantitative value of the samples/(sum of the quantitative value of the samples/sum of the quantitative value of QC1). Compounds whose coefficient of variation (CV) of the relative peak areas in the QC samples was greater than 30% were removed. Metabolites were annotated via the KEGG database (https://www.genome.jp/kegg/ pathway.html), HMDB (https://hmdb.ca/metabolites) and LIPIDMaps database (http://www.lipidmaps. org/). Principal component analysis (PCA) was performed via the metaX R package. We applied univariate analysis (t test) to calculate the statistical significance (P value), and a P value < 0.05 was considered statistically significant. FC refers to the fold change, which is the ratio of the mean of 6 biologically repeated quantitative values of each metabolite in the comparison group. The differentially abundant metabolites were screened via the variable importance in projection (VIP), P value and FC. The differentially abundant metabolites are listed in Supplementary Table 4. Volcano plots were used to filter metabolites of interest on the basis of the log2 (fold change) and -log10 (p value) values of the metabolites via ggplot2 in the R language. For clustering heatmaps, the data were normalized via z scores of the intensity areas of differentially abundant metabolites and plotted via the Pheatmap package in R language.

## Supplementary Information

Supplementary Information 1.

Supplementary Information 2.

Supplementary Information 3.

Supplementary Information 4.

Supplementary Information 5.

## Data Availability

All data generated or analyzed during this study are included in this manuscript or its supplementary files. The transcriptome raw data have been deposited in the NCBI Gene Expression Omnibus (GEO) database under accession number GSE318428.
