# Supplementary material for: Integrated transcriptomic and metabolomic analyses reveal distinct energy metabolic signatures and functional properties of RPE cells under two culture conditions
Source: Sci Rep. 2026 Apr 10;16:11992. doi: 10.1038/s41598-026-39689-9 (PMC13068893; doi:10.1038/s41598-026-39689-9)
Supplement: Supplementary file 1 — Supplementary Information 1. [file 41598_2026_39689_MOESM1_ESM.docx]

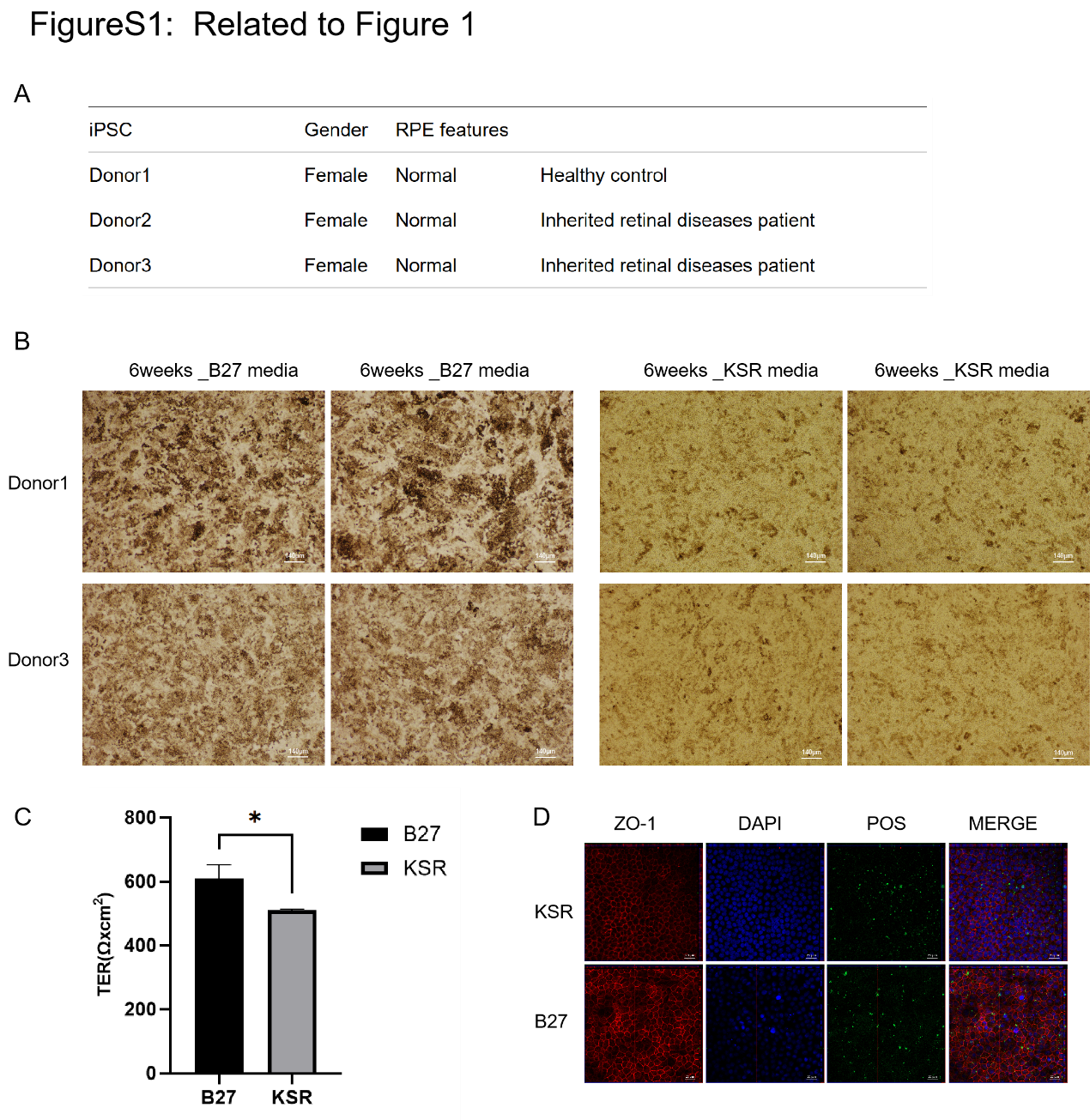


Figure S1: Related to Figure 1
A: Donors of iPSC used to test the two different culture media, the iPSC of donor1 was used in this study; B: Morphology and pigmentation of RPE derived from 2 iPSC lines ;C: TER measurements of RPE from donor3 cultured for 4 weeks, *P<0.05, using unpaired t-tests; N=3 per group. Values are expressed as means±SEM; D: Representative images showing phagocytosis of photoreceptor outer segments (POS) by RPE cells；


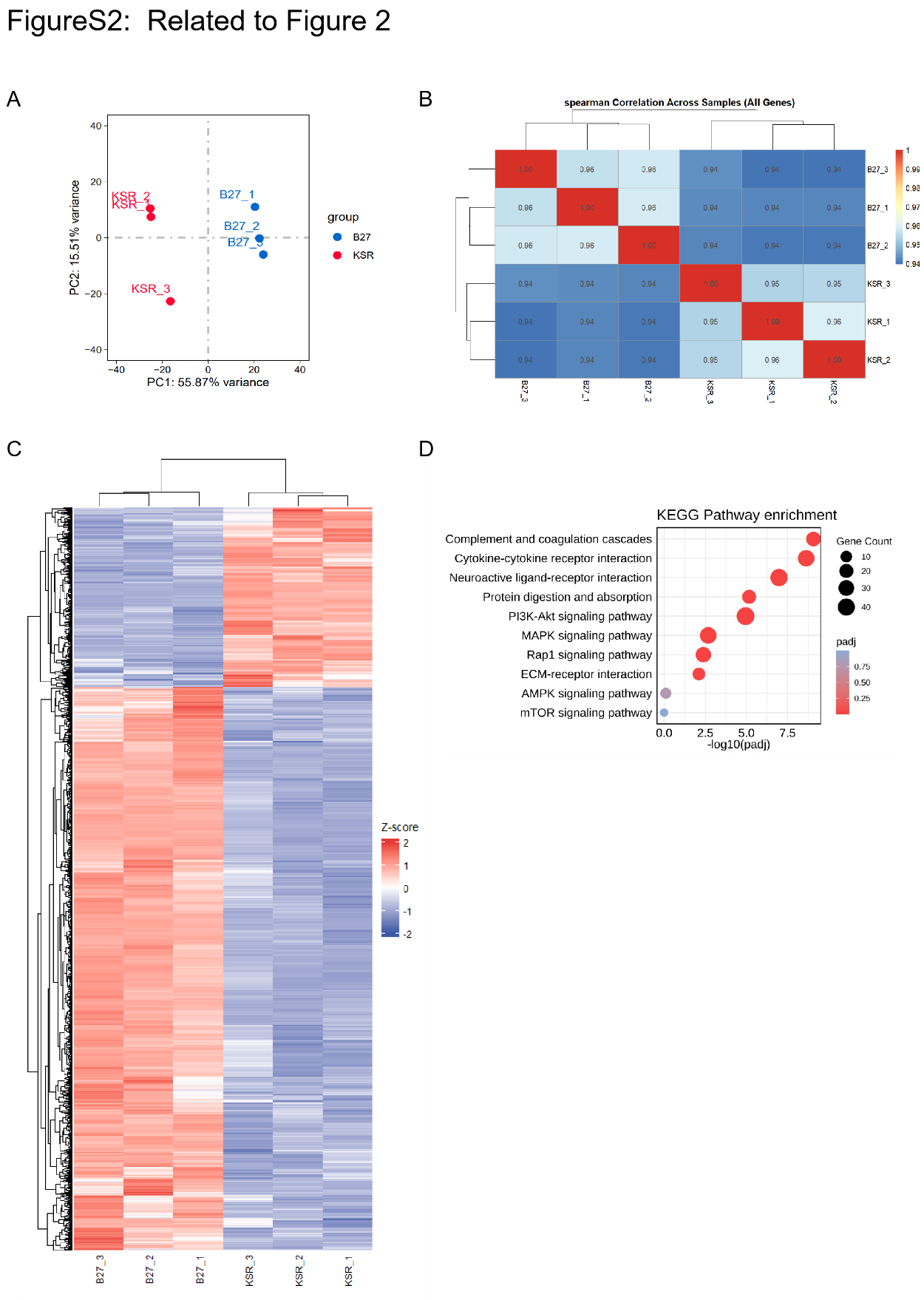


Figure S2: Related to Figure 2
A: Principal component analysis (PCA) of transcriptomic data of 4-week cultured KSR- and B27-RPE cells; B: Spearman correlation analysis of KSR- and B27-RPE cells based on all expressed genes；C: Heatmap of all differentially expressed genes of KSR- and B27-RPE cells; D: KEGG enrichment analysis^1, 2, 3^ of all differentially expressed genes of KSR- and B27-RPE cells.


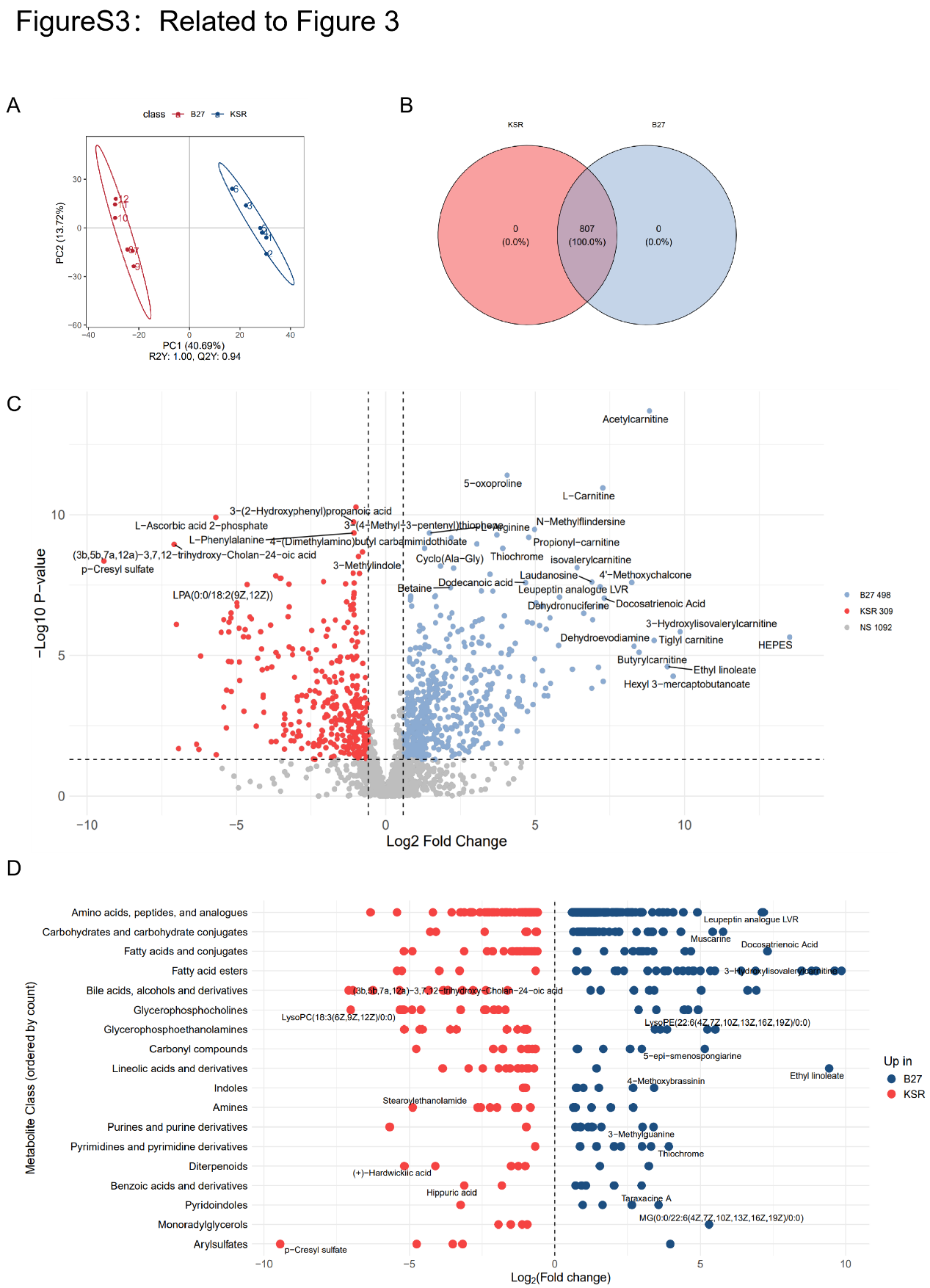


Figure S3: Related to Figure 3
A: Principal component analysis of untargeted metabolomic profiles of 4-week cultured KSR- and B27-RPE cells; B: The distribution of metabolites between the two groups; C: Volcano plot of differentially abundant metabolites between the two groups; D: Classification of the differential metabolites according to Class III metabolite categories.


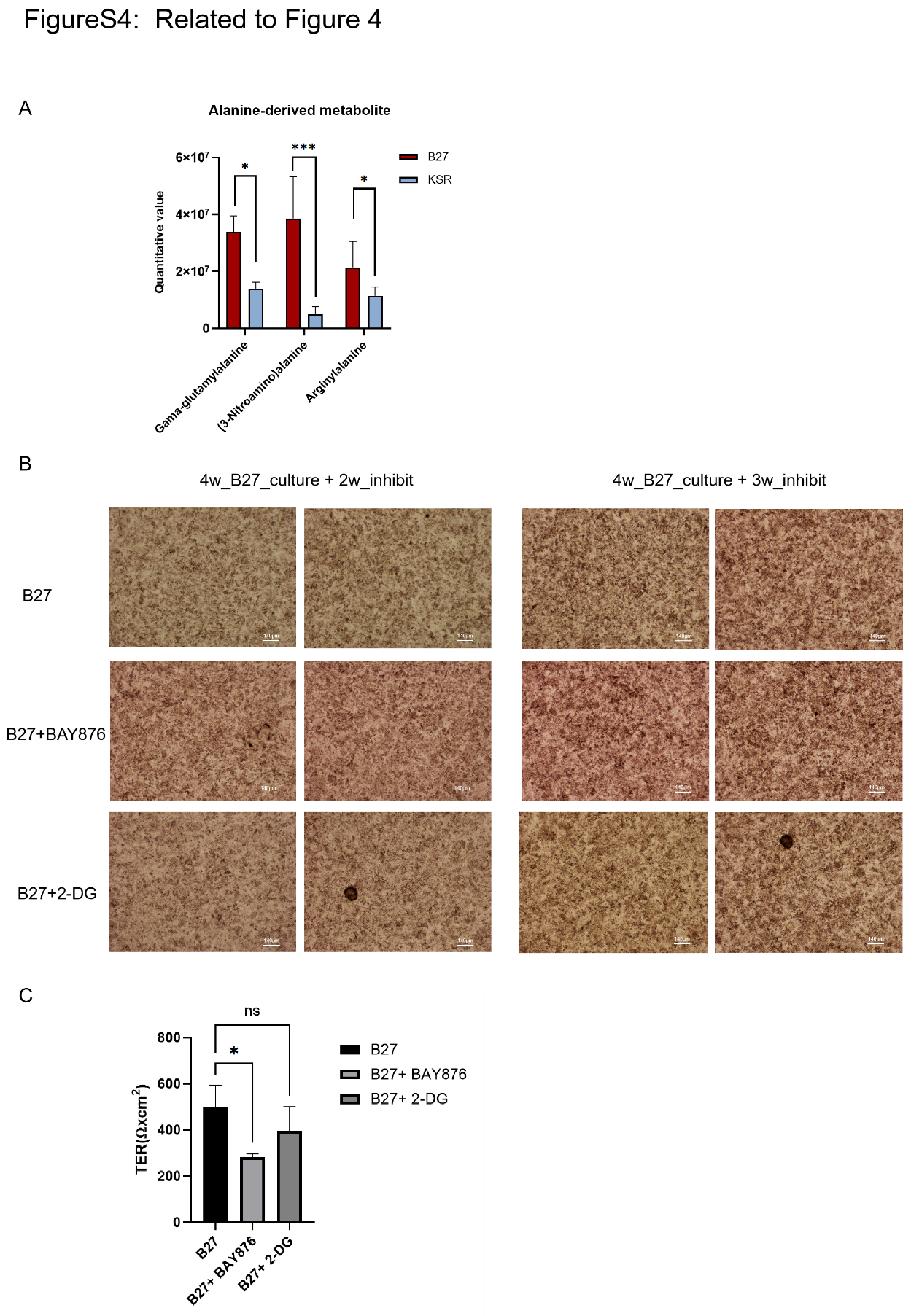


Figure S4: Related to Figure 4
A: Quantitative value of alanine-derived metabolites in B27and KSR RPE cells; B: Morphology of control, BAY876 inhibited and 2-DG inhibited B27-RPE for 2 and 3 weeks; C: Measurement of TER of control, BAY876 inhibited and 2-DG inhibited B27-RPE for 3 weeks;

**References**

1. Kanehisa MaG, Susumu KEGG: Kyoto Encyclopedia of Genes and Genomes. *Nucleic Acids Research*, (2000).

2. Kanehisa M. Toward understanding the origin and evolution of cellular organisms. *Protein Science* **28**, 1947-1951 (2019).

3. Kanehisa M, Furumichi M, Sato Y, Matsuura Y, Ishiguro-Watanabe M. KEGG: biological systems database as a model of the real world. *Nucleic Acids Research* **53**, D672-D677 (2025).
